# A Generalized and Real-Time Network Intrusion Detection System Through Incremental Feature Encoding and Similarity Embedding Learning

**DOI:** 10.3390/s25164961

**Published:** 2025-08-11

**Authors:** Zahraa kadhim Alitbi, Seyed Amin Hosseini Seno, Abbas Ghaemi Bafghi, Davood Zabihzadeh

**Affiliations:** 1 Computer Engineering Department, Engineering Faculty, Ferdowsi University of Mashhad (FUM), Mashhad 91779-48974, Iran; zahraa.alitbi@mail.um.ac.ir (Z.k.A.); ghaemib@um.ac.ir (A.G.B.); 2 Computer Engineering Department, Hakim Sabzevari University, Sabzevar 96179-76487, Iran; d.zabihzadeh@hsu.ac.ir

**Keywords:** network intrusion detection, novel attack detection, real-time intrusion detection, incremental learning, transformer model, semantic embedding learning

## Abstract

Many *Network Intrusion Detection Systems* (NIDSs) process sessions only after their completion, relying on statistical features generated by tools such as CICFlowMeter. Thus, they cannot be used for real-time intrusion detection. Packet-based NIDSs address this challenge by extracting features from the input packet data. However, they often process packets independently, resulting in low detection accuracy. Recent advancements have captured temporal relations between the packets of a given session; however, they use a fixed window size for representing sessions. This representation is inefficient and ineffective for processing short and long sessions. Moreover, these systems cannot detect unobserved attack types during training. To address these issues, the proposed method extracts features from consecutive packets of an ongoing session in an online manner and learns a compact and discriminative embedding space using the proposed multi-proxy similarity loss function. Using the learned embedding and a novel class-wise thresholding approach, our method alleviates the imbalance issue in NIDSs and accurately identifies observed and novel attacks. The experiments on two large-scale datasets confirm that our method effectively detects attack activities by processing fewer than seven packets of an ongoing session. Moreover, it outperforms all the competing methods by a large margin for detecting observed and novel attacks.

## 1. Introduction

*Network Intrusion Detection Systems* (NIDSs) play a crucial role in securing computer networks by monitoring network traffic for detecting malicious activities. The majority of NIDSs manage traffic by organizing it into logical units called *sessions*. These systems store all traffic within a session, extract statistical features from the traffic, and utilize them to discriminate intrusions from normal sessions [1,2,3].

A session in *Transmission Control Protocol* (TCP) contains a *forward flow* (source → destination) and a *backward flow* (destination → source). *Inter-Arrival Time* (IAT) of two adjacent packets is a key indicator in determining the end of a flow in different network protocols, such as TCP, *User Datagram Protocol* (UDP), and *Internet Control Message Protocol* (ICMP). If *IAT* surpasses a predefined threshold, the flow is terminated. However, even in TCP connections, packet retransmissions due to congestion or packet loss are common. Therefore, the maximum IAT should be set high enough to handle such scenarios. As a result, session-based NIDSs cannot be used for the early detection of network intrusions.

To address this issue, *packet-based* NIDSs have been introduced, which extract features from each packet’s data [4,5]. Many packet-based methods examine each packet independently, leading to poor detection accuracy and high false alarm rates. Some methods address this issue by considering the temporal relationships between packets in a session; however, they rely on a fixed window size to represent the session. This static window size is inefficient for short sessions and ineffective for long ones.

Another significant challenge in NIDSs is the detection of *novel and mutated* attacks. *Signature-based* NIDSs struggle to detect zero-day attacks due to relying on predefined attack patterns. Moreover, many multi-class *anomaly-based* NIDSs classify input traffic into observed classes during training and cannot identify unseen attack types.

The proposed method, named the *Generalized and Real-time IDS* (GR-IDS), addresses these two main challenges in NIDSs: (1) early detection of network intrusions using only initial packets of a session with high performance, and (2) detection of novel or mutated attack types.

To this end, GR-IDS presents an *incremental* feature encoding module that automatically extracts features from M consecutive packets of an ongoing session in an incremental manner.

The module combines a packet-window transformer with a session-level LSTM state to realize a multi-scale model. The transformer effectively extracts discriminative features from the current sequence by capturing the relationships among the packets of the sequence. The LSTM mines the time-related information between the observed sequences of the session. Simultaneously, our method incrementally computes statistical features of the current session. By combining statistical features with the discriminative features extracted by the transformer–LSTM model, GR-IDS can detect intrusion sessions early with high performance.

Additionally, inspired by successful applications of deep metric learning in open-set recognition, GR-IDS learns a semantic embedding space to identify intrusions. Specifically, it learns multiple proxies for each attack category to capture its variants effectively in the embedding space. Furthermore, we introduce a loss term that creates a compact representation for each class, which not only improves the classification accuracy of known classes but also helps in identifying novel attacks.

Finally, GR-IDS alleviates the *imbalance* issue in NID by learning similarity-based embedding to better represent the data distribution and a *novel class-wise thresholding* approach for classifying incoming packet sequences.

We evaluated the performance of GR-IDS through experiments conducted on two large-scale datasets, namely CIC_DDoS2019 [6] and CRiSIS-2022 [7]. The results show that GR-IDS accurately detects observed and novel attack types with low false alarm rates by processing only initial packets of any input session.

The structure of this paper is as follows: Section 2 discusses related work in network intrusion detection. Section 3 presents the model, training procedure, and attack detection process of the proposed method. Section 4 presents experimental results, comparing GR-IDS with existing methods and analyzing the impact of individual components through an ablation study. Lastly, Section 5 summarizes the key findings and introduces potential directions for future research.

## 2. Related Work

This section reviews session-based and packet-based approaches for developing NIDSs. Afterward, it discusses recent methods for detecting novel attack types.

### 2.1. Session-Based Methods

The dominant approach for developing NIDSs is to extract information from sessions and train ML models to detect intrusions. Available tools such as CICFlowMeter [8] can generate session features from packet header data.

In addition to traditional ML models, deep neural networks such as *Convolutional Neural Networks* (CNNs), *Auto Encoders* (AEs), and *Recurrent Neural Networks* (RNNs) have been utilized for building NIDSs. For example, Refs. [9,10] used *deep learning* (DL) models for detecting intrusions and compared their performance with several ML models.

Some methods [11,12,13] used AE models to encode session features and then utilized ML models such as *Random Forest* (RF) and *Support Vector Machine* (SVM) to identify intrusions using the encoded features.

Some studies [14,15,16,17] have used RNN models for developing NIDSs. Yin et al. [14] trained an RNN model for multi-class classification of sessions in the NSL-KDD dataset. The model outperformed ML models on this dataset. Xu et al. [15] developed an NIDS using *Gated Recurrent Units* (GRUs). However, the NIDS achieved lower accuracy for detecting minority attack types.

Zavrak et al. [17] utilized a *Long Short-Term Memory* (LSTM) model to detect attacks using session features generated by OpenFlow switches.

Some methods [18,19] have utilized hybrid deep neural networks to improve the performance of NIDSs. For example, Hnamte et al. [19] combined a CNN with a *bidirectional* LSTM.

To improve the generalization of NIDSs across different datasets, Refs. [20,21] only used NetFlow features to develop NIDSs.

Several studies [22,23,24,25,26,27,28] have shown that deep metric learning losses, such as triplet and pairwise loss functions, can effectively mitigate the class imbalance issue in NIDSs. Bedi et al. [24] accurately identified minority intrusion classes using an *under-sampling* strategy, pairwise loss, and a *Siamese* network. For example, Ref. [24] achieved recall rates of 33.25% and 56.72% for detecting R2L and U2R minority classes on NSL-KDD, whereas a baseline CNN model trained using cross-entropy loss only achieved recall rates of 6.44% and 17.91%, respectively, on these classes.

Andresini et al. [27] developed an NIDS using AEs and triplet losses. This method considered one AE for each class and enforced each AE to reconstruct the examples of the corresponding class accurately, while reconstructing instances of other classes poorly. The results in [27] confirm that this method significantly improves the performance of DML-based methods. For example, Ref. [27] achieved 98.24% accuracy and a 95.27% F1-score for binary intrusion detection on CIC-IDS2017 [8], while a triplet network trained using random triplet sampling and the kNN classifier in the predictive stage only achieved 58.63% accuracy and a 27.33% F1-score.

Although the performance of many session-based methods is promising, they classify an ongoing session once it is completed; thus, they cannot be used for early (near-real-time) intrusion detection.

### 2.2. Packet-Based Methods

An alternative approach for developing NIDSs is to directly extract features from raw packets. Qin et al. [29] presented an RNN model with an attention mechanism to mine features from the payload of an incoming packet. They evaluated the model for binary intrusion detection on the CIC-IDS2017 dataset. Farrukh et al. [30] provided a tool named payload-byte to extract packet payloads from *packet capture* (pcap) files. They trained deep neural networks using the extracted payloads. Their experiments indicate that the deep models can obtain competitive performance with session-based NIDSs. Zhang et al. [31] transformed a given packet payload into a grayscale image and then used the inverse discrete cosine transform to extract features from the image.

These methods [29,30,31] only extracted features from packet payloads. However, some attacks like *distributed denial-of-service* (DDoS) target packet headers. Moreover, they extracted features from each packet separately and did not mine the temporal and spatial relations between packets of a session.

Yu et al. [32] transformed packets of an input session into a grayscale image. Similarly, Ghadermazi et al. [33] generated a colored image for each input session, in which the red channel stores packet data of the forward flow and the green channel stores packet data of the backward flow. These methods [32,33] then learned discriminative features from the image using CNNs. The results showed that they achieved an accuracy and F1-score of about 98% on CIC-IDS2017, outperforming the peer PayloadEmbeddings method [34] by around 3%.

However, these methods use a fixed image size for representing a session. This representation wastes a large amount of memory for encoding short sessions. Moreover, it is ineffective for processing long attack sessions in which the initial packets do not contain malicious patterns. Finally, these methods cannot identify unseen attacks.

Han et al. [1] used a two-layer LSTM model to extract features from an input packet in an online manner. This method partially classified an ongoing session while receiving a new packet. However, classifying the traffic each time an individual packet is received demands a packet processing rate of up to 30 million packets per second to support 10 Gbps traffic, which is an extremely challenging requirement, even for dedicated hardware accelerators [1].

Pekar et al. [35] experimentally found that the performance of ML models such as RF is significantly reduced by up to 30% when they are trained on complete sessions and are tested against incomplete sessions. Moreover, models trained on partial sessions can maintain their robust performance for classifying partial sessions during the test stage. The study also showed that RF models require a minimum of seven packets for each session on CIC-IDS2017 to achieve a high and stable detection rate.

### 2.3. Detecting Novel Attack Types

Zero-shot learning and few-shot learning are two common approaches for identifying novel attacks. *Zero-shot learning* (ZSL) aims to detect new attacks using only their semantic descriptions. To this end, models should learn mappings between the feature space and semantic spaces. Refs. [36,37] have utilized ZSL and AE models to detect new intrusions on the NSL-KDD [38] and NF-UNSW-NB15-v2 [39] datasets. The results in [36] showed that the ZSL method improved the recall and precision of detecting zero-day attacks by about 2% on NSL-KDD [38] compared to the AE model.

Similarly, Ref. [40] employed ZSL to identify novel attacks on the *Segmented Intrusion Detection Dataset* (SSIDD) [41], an image-based intrusion detection dataset. ZSL needs semantic descriptions for novel attacks; however, this information is unavailable once these attacks occur. Moreover, these methods [36,37,39] are session-based and cannot be used for early intrusion detection.

Refs. [42,43] have employed *few-shot learning* (FSL) to identify novel attacks. For example, Wang et al. [42] used a clustering and sampling algorithm to obtain a balanced training set and then applied FSL. FSL needs examples from all classes during the training stage; however, acquiring even a small number of unknown attack types is infeasible in practice.

## 3. Materials and Methods

This section presents *GR-IDS*, including its incremental feature encoding pipeline, its semantic embedding module, and the learning objective that yields compact, discriminative embeddings suited to early intrusion detection. It also provides pseudo-code for our method and describes the proposed novel attack detection strategy.

### 3.1. Feature Encoding

To address the main issues of session-based NIDS methods, we design a feature encoder that automatically extracts features from M *consecutive* packets of a session in an incremental manner, thus eliminating the need to store incoming packets. The proposed feature encoder consists of two modules: (1) *a packet data feature extractor* and (2) *an online statistical feature calculator.*

The former is a hybrid deep model that integrates a transformer model and an LSTM model. In real-time settings, pure transformers require a large window proportional to sequence length, which burdens GPU memory and complicates online inference. However, by constraining the attention window and letting an LSTM maintain session state, we bound memory while retaining long-range dependencies. Specifically, if $d_{T}$ denotes the packet feature dimension, the attention mechanism on window M requires $O\;(M^{2}d_{T})$ operations while pure transformer over the full session with length L≫M needs $O\;(L^{2}d_{T})$ operations. By appending an LSTM that maintains session state, we restore long-range temporal dependencies with $O\;(M\;d_{T}^{2})$ incremental cost and *constant memory* with respect to L.

**Figure 1:**
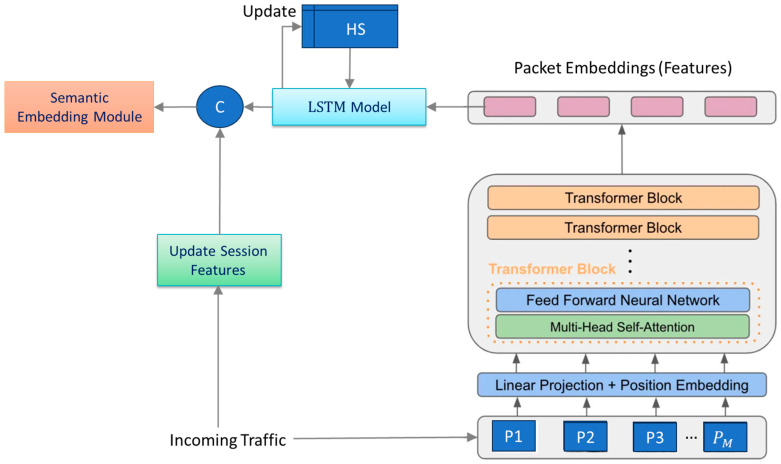
Overall structure of the proposed deep feature encoder model.

The latter module updates the *session-level statistics* after receiving a new packet using *the recurrence relations*, similarly to [1]. Figure 1 shows the overall structure of the proposed feature encoder.

The incoming packets are processed as in [33]. Specifically, we remove the 14-byte Ethernet header plus IP/TCP control fields (version, description, protocol, IP options, TCP options, source/destination IP and ports) to avoid misalignment and reduce noise. Only the first 100 bytes of packet data are kept (shorter packets are 0-padded). Each byte is mapped to 0,1 via min–max scaling and the resulting 100-D vector p is $L_{2}$-normalized, ensuring that ${\left\|p\right\|}_{2}=1.$

Let $x=\left(p_{1},\ldots ,p_{M}\right)\in R^{Md}$ be the input sequence including M consecutive packets. This sequence is processed by the transformer model, denoted by $f\left(.\right)$, to extract discriminative features that capture the relationships among these packets. To keep the window size M small while maintaining high performance for classifying long sessions, the LSTM network maintains a hidden state HS that is reinitialized at the start of every session. HS summarizes all sequences processed so far and therefore encodes higher-level temporal dependencies across the session.

#### 3.1.1. Transformer Model

As shown in Figure 1, the transformer model consists of multiple blocks, each containing a *multi-head self-attention* module, ${MH}_{A}tt(.)$, and a small *feed-forward neural network* $F\left(.\right)$. Before the data enters these blocks, a linear layer projects the input dimensions to the required dimension $d_{T}$.

Moreover, since the multi-head attention module treats inputs as an unordered set, but the order of packets is crucial in NIDSs, we use *positional encoding* to encode positional information into the inputs. We employ the technique proposed by [44], which uses sine and cosine functions of different frequencies for encoding a position pos in the input features at dimension i as follows:

(1) $$PE\left(pos,\;i\right)\;=\left\{\begin{array}{c} \sin\left(\frac{pos}{1000^{i/d_{T}}}\right),\;\;\;\;\;\;\;\;\;\;\;\;\mathrm{if}\;i\;\mathrm{is}\;\mathrm{even},\\ \cos\left(\frac{pos}{1000^{\left(i-1\right)/d_{T}}}\right),\;\;\mathrm{otherwise.} \end{array}\right.$$

Here, $PE\left(pos,\;i\right)$ denotes the position encoding at position pos (1<pos<M) in the sequence for dimension i ($1\leq i\leq d_{T}$). These values, concatenated across all dimensions, are then *added* to the projected packet features before the first transformer block.

**Figure 2:**
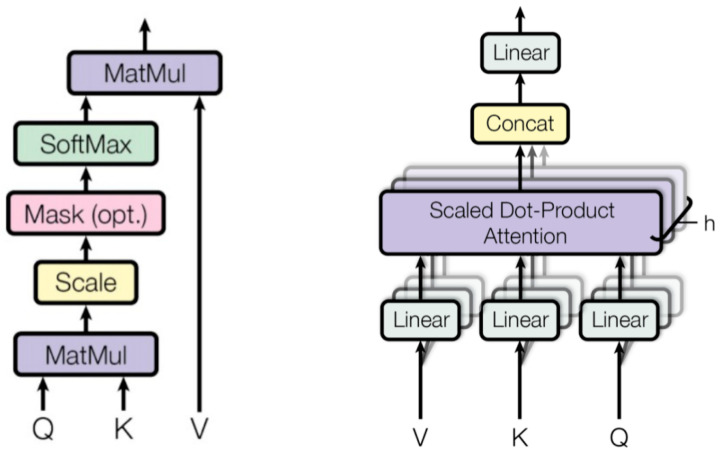
**Left**: The architecture of the scaled dot-product attention (self-attention). **Right**: The architecture of the multi-head attention module.

Figure 2 illustrates the architecture of the multi-head self-attention module. At the core of the multi-head attention module is the self-attention mechanism. For any element p, self-attention generates a weighted average of the elements in the sequence, where the weights are computed dynamically based on the similarity between p and each of the other elements. Obtaining multiple distinct attention features from an input sequence often improves model performance. Hence, transformer models apply multiple self-attention heads in each block.

Within each transformer block, the output of the multi-head attention is passed to a small, fully connected feed-forward network $F\left(.\right)$ implemented by two fully connected (FC) layers and a ReLU activation function (FC→ReLU→FC). The entire transformation in each block to an input x can be expressed as

(2)     x⟵x+dropout(MH_Att(x))x⟵Layer_norm(x)   x⟵Layer_norm(x+F(x))

As observed, residual connections are used to form the transformer blocks, and the output of each block has the same dimension as the input.

#### 3.1.2. LSTM Network

This network maintains the hidden state of the current session HS. Let the ${HS}^{\left(t-1\right)}$ denote the hidden state of the current session at timestep (t−1), which is initialized with random values. By receiving x at timestep t, if x represents a new session, ${HS}^{\left(t-1\right)}$ is reinitialized. Then, the network processes x as

(3) $$f_{L}^{\left(t\right)},{HS}^{\left(t\right)},⟵LSTM\left(x,\;{HS}^{\left(t-1\right)}\right),$$

where $f_{L}^{\left(t\right)}$ is the output (extracted features) of the network and ${HS}^{\left(t\right)}$ denotes the updated hidden state summarizing all past information from the current session up to time t.

#### 3.1.3. Online Statistical Flow Feature Extractor

This module computes a set of statistical features for each active session, as listed in Table 1. To support real-time computation, all variables marked as *State* in Table 1 are incrementally updated and stored for every ongoing session. Here, t and l show the timestamp and packet length (in bytes) of a newly arrived packet in the current session f.

In addition to each variable listed in Table 1, we also consider two corresponding variables for the forward and backward flows of each session. For example, in addition to pkt_rate, we define fwd_pkt_rate and bwd_pkt_rate, representing packet rates in the forward and backward directions, respectively.

This module updates the state of session f and recomputes the relevant statistics according to the equations in Table 1. If the packet direction is forward, then the forward-related variables of f are updated following the same equations. Otherwise, the backward-related variables are updated similarly.

Subsequently, the statistical features of f are normalized to have zero mean and unit standard deviation and then stored in the feature vector $f_{S}$, which is passed to the feature fusion module.

#### 3.1.4. Feature Fusion

This module first linearly transforms the feature vector $f_{L}$ to ensure that its dimensionality matches that of the statistical vector $f_{S}$. The two vectors are then concatenated and passed to the semantic embedding module for further processing.

### 3.2. Semantic Embedding Learning

This section introduces the semantic embedding module that maps encoded features to a compact and discriminative embedding space and learns multiple proxies per class to capture intra-class variations.

#### 3.2.1. Architecture

The semantic embedding module consists of a projection head g, a proxy layer, and a similarity-based loss function. Let $f\in R^{d_{f}}$ be the input feature vector. The projection head consists of two fully connected layers with a ReLU activation applied after the first layer. It transforms f into a low-dimensional embedding $z\in R^{d_{e}}$. Afterward, a Layer_norm is applied to z, ensuring that ${\left\|z\right\|}_{2}=1.$

In high-dimensional spaces, Euclidean distance becomes less meaningful due to the curse of dimensionality; thus, projecting features into a lower-dimensional space improves the effectiveness of the proposed similarity-based loss. Moreover, a smaller embedding dimension reduces model complexity and prevents overfitting.

Attacks in NIDSs have different variations. For example, DDoS attacks include variations such as *synchronized* (SYN) flooding, UDP flooding, and ICMP flooding. To capture the underlying distribution of the traffic inside each class more effectively, we consider multiple proxies for the class. These proxies are trained simultaneously with the other model parameters using a *backpropagation-based optimizer* like Adam [45].

#### 3.2.2. Loss Function

For a natural number n, let $\left[n\right]$ denote $\left\{1,2,\ldots ,n\right\}.\;$The set of proxies for each class $c\in \left[C\right]$ is denoted by ${\left\{w_{j}^{c}\right\}}_{j\in \left[J\right]}$. We define the similarity between the embedding z and class c, $s\;\left(z,c\right)$ as follows:

(4) $$s\left(z,c\right)=\sum_{j\in \left[J\right]}\alpha_{j}^{c}z^{⊤}{\tilde{w}}_{j}^{c},$$

where ${\tilde{w}}_{j}^{c}=w_{j}^{c}/{\left\|w_{j}^{c}\right\|}_{2}$, and $\alpha_{j}^{c}$ indicates the attention weight of proxy $w_{j}^{c}$ obtained by the self-attention mechanism. Specifically, we treat z as a query, while treating all proxies ${\left\{w_{j}^{c}\right\}}_{j\in \left[J\right]}$ as both keys and values. The attention weight $\alpha_{j}^{c}$ is computed by Softmax over the similarity scores between z and each $w_{j}^{c}$.

The similarity $s\left(z,c\right)$ lies within the $\left[-1,+1\right]$ range, because z and ${\tilde{w}}_{j}^{c}$ have a unit $l_{2}\text{-}norm$, $\alpha_{j}^{c}\geq 0$, and $\sum_{j\in \left[J\right]}\alpha_{j}^{c}=1$. The embedding’s prediction for z, $p=\left(p_{1},\ldots ,p_{C}\right)$ is obtained by applying Softmax to the similarities as follows:

(5) pc=p y=c|z=exp sz,c/T∑i∈Cexp s z,i/T,c∈C,

where T indicates the Softmax temperature.

The proposed loss function is a regularized cross-entropy loss between the embedding’s prediction p and the true label y of z:

(6) $$L_{e\;}\left(z,y\right)=-\log p_{y}+\lambda_{r}{\left\|\alpha^{y}\right\|}_{1},$$

where $p_{y}$ indicates the predicted probability of the target class y, and the regularization term encourages the model to generate sparse attention weights, thus increasing the confidence of the predictions within the target class. The hyperparameter $\lambda_{r}$ controls the weight of the regularization term.

#### 3.2.3. Compactness Loss Term

Instances of the same class may form large clusters in the learned embedding space, leading to high intra-class variation. To improve the performance in classifying observed classes and effectively identifying novel attacks, we introduce a loss term $\left(L_{c}\right)$ that minimizes the distance between each embedding z and its corresponding class y, while simultaneously separating z from other classes, resulting in a more compact representation of the class. $L_{c}$ is defined as

(7) $$L_{c}\;\left(z,y\right)=\max\;\left\{m-s\;\left(z,y\right),0\right\}+\sum_{i\in \left[C\right]\backslash \{y\}\;}\max\left\{s\;\left(z,i\right)-m,0\right\},$$

where m denotes a margin hyperparameter.

### 3.3. Final Loss and Training Algorithm

The final loss function in GR-IDS is given as

(8) $$L\left(z,y\right)=L_{e}\;\left(z,y\right)+\lambda_{c}L_{c}\;\left(z,y\right),$$

where the hyperparameter $\lambda_{c}$ controls the trade-off between the loss terms. Algorithm 1 shows the main training steps of GR-IDS.
**Algorithm 1**. Main Training Steps of GR-IDS**Input**: $D\;=\;{\left\{(p_{i},\;y_{i})\;\right\}}_{i=1}^{n}$: a sequence of incoming labeled packets **Output**: Trained IDS model (the transformer, LSTM, and embedding modules)Initialize a pool of session states to maintain statistical information for active sessions.Initialize a pool of session hidden states to maintain packet sequence information for active sessions.**for** iter = 1, 2, …, MAX_Iter **do**: Receive an incoming packet and update its corresponding session information according to Table 1. **if** a session is terminated or M subsequent packets of a session are received **then**:  
$f_{s}$ = Retrieve statistical features of the session  
HS = Retrieve hidden state of the session  
$f_{L}$  = Transformer-LSTM.forward(x , HS)  
f $\;=\;\mathrm{Combine}(f_{s}$ $,\;f_{L}$)  
z  = g(f)  
s = Calculate similarities between z and all classes using Equation (4)  
p = Calculate embedding’s prediction using Equation (5)  
L = Compute the loss using Equations (6)–(8)  Backpropagate L to update the model parameters **end if****end for****return** Trained IDS Model

### 3.4. Novel Attack Detection

Let $x=\left(p_{1},\ldots ,p_{M}\right)\in R^{Md}$ represent the input sequence of M consecutive packets from the current session f. First, the feature vector of x is extracted and its embedding z is obtained. Afterward, the embedding’s prediction for z is computed using Equations (4) and (5). Let $\tau_{c}$ be the specified threshold for class c. The set of candidate classes C′ for z is defined as

$$C^{\prime }=\left\{c|\;p\left(y=c|z\right)\geq \tau_{c}\right\}$$

If $C^{\prime }$ is empty, the session is labeled as a novel attack. Otherwise, the class $\hat{c}\in C^{\prime }$ with the maximum value of $p\;\left(y=c|z\right)$ is chosen: $\hat{c}=arg\underset{c}{\max}\left\{p\;\left(y=c|z\right)|c\in C^{\prime }\right\}.$

The threshold of any class c is adjusted using validation data. Specifically, to adjust $\tau_{c}\in \left[C\right]$, we compute Youden’s J statistic [46], defined as $J_{c}=\mathrm{Sensitivity}+\mathrm{Specificity}-1$, on the validation set for class c. Then, $\tau_{c}$ is set as the threshold value which maximizes $J_{c}$. Maximizing $J_{c}$ reduces the risk of false alarms (low specificity) while maintaining high detection performance (high sensitivity).

It also mitigates the imbalance issue in NIDSs because by employing per-class thresholds optimized via Youden’s J statistics, classes with more data (e.g., the normal class) tend to have stricter thresholds. Suppose that the normal class is the majority and yields the highest similarity score for x, while x belongs to an attack class. Since the threshold of the normal class is set higher, the likelihood of $p\;\left(y=normal|z\right)$ being below $\tau_{normal}$ is higher. In that case, only attack classes will appear in the candidate set.

### 3.5. Memory and Time Complexity

Our feature encoder combines a packet-window transformer with a session-level LSTM to realize a multi-timescale model. As shown in Table 2, the transformer and LSTM models have $O\;(Mdd_{T}\;+d_{T}^{2})$ and $O\;(Md_{T}^{2})$ learnable parameters, respectively. Additionally, the feature fusion module is a compact component, adding only $O\;(d_{T}d_{f})=O\left(d_{T}^{2}\right)$ parameters.

Since $d_{T}<\;d$, the memory requirement of the feature encoder will be $O(Mdd_{T})$. Using the experimental settings ($i.e.,\;d=100,\;d_{T}=32,\;and\;M=4,\;d_{f}=48$), the encoder includes approximately 27,040 learnable parameters, which is a relatively small number compared to typical deep learning architectures.

The proposed semantic embedding module has a simple architecture, including $O(d_{f}d_{e}\;+\;d_{e}\;C\;|J|)$ parameters. For example, in the CIC_DDoS2019 dataset (C=13), when $\left|J\right|=5,\;d_{f}=48,\;and\;d_{e}=32$ (the settings used in the experiments), it involves only 3648 learnable parameters.

In terms of computational complexity, projecting input to the model dimension $d_{T}$ demands $O\;(Mdd_{T})$ operations. The transformer’s attention mechanism on window M requires $O\;(M^{2}d_{T})$ operations and LSTM adds $O\;(Md_{T}^{2})$ computations with *constant memory* with respect to session length. Subsequently, classifying the input window by the semantic embedding module involves $O\;\left(d_{f}\;d_{e}\;+\;d_{e}\;C\;\left|J\right|\right)=O\;\left(d_{T}^{2}+d_{T}C\;\left|J\right|\right)$ operations.

Since $d_{f}\;and\;d_{e}$ are proportional, and $d_{T}\;$and $C\;\left|J\right|<d$, the overall time complexity for the model’s prediction will be

$$t\;\left(M,\;d,d_{T}\right)=O\;\left(Mdd_{T}+M^{2}d_{T}+Md_{T}^{2}+d_{T}^{2}+d_{T}\;C\;\left|J\right|\right)=O\;\left(Mdd_{T}\right).$$

Consequently, the most computationally intensive step in the encoder is projecting the packet input features into the model’s lower-dimensional space. Thus, the model can process packet sequences with low latency.

## 4. Results

This section discusses the experiments conducted to evaluate the performance and efficiency of GR-IDS for detecting observed and novel attacks. We compare the proposed method with several state-of-the-art baselines. Moreover, we investigate the contribution of different modules and mechanisms in the proposed method through an ablation study. Finally, we analyze the sensitivity of GR-IDS with respect to several key hyperparameters.

### 4.1. Datasets

The proposed method is evaluated on two standard datasets: CIC_DDoS2019 [6] and CRiSIS-2022 [7]. Table 3 provides the specifications of these datasets.

CIC_DDoS2019 was recorded over two days of network activity. For each day, packet sequences, including payloads, are available in *pcap* format. It also contains 80 flow features extracted from pcap files. The dataset includes benign traffic and twelve different DDoS attack types.

CRiSIS-2022 extends the CIC-IDS2017 dataset [8] by resolving some significant labeling errors and flaws in capturing network traffic. It contains 2,830,743 flows, including 14 different attacks and normal traffic. Each flow is described by 79 features. The dataset also provides the raw pcap files with packet-level details.

### 4.2. Evaluation Metrics

We utilize the standard classification criteria to evaluate the performance of GR-IDS. Let C and n denote the number of classes and sessions in the test set, respectively. We assume that the ID of the normal class is zero. Moreover, let $n_{ij}$ denote the number of instances in class i that are classified as class j ($i,j\in S=\left\{0,1,\ldots ,C-1\right\}$), and let $n_{i}=\sum_{j\in S}n_{ij}$ denote the number of sessions in class i. Then, the classification metrics are defined as follows:

(9) $$Accuracy=\frac{\sum_{i\in S}n_{ii}}{n},$$

(10) $$Precision\left(i\right)=\frac{n_{ii}}{\sum_{j\in S}n_{ji}},$$

(11) $$Recall\left(i\right)=\frac{n_{ii}}{n_{i}}$$

(12) $$\mathrm{Macro}\mathrm{Accuracy}=\frac{\sum_{i\in S}Recall\left(i\right)}{C}$$

(13) $$WeightedPrecision\;\left(WPrecision\right)=\frac{\sum_{i\in S}n_{i}\;Precision\left(i\right)}{n},$$

(14) $$WeightedRecall\;\left(WRecall\right)=\frac{\sum_{i\in S}n_{i}\;Recall\left(i\right)}{n},$$

(15) $$WeightedF1\text{-}Score\;\left(WF1\right)=\left(\frac{2}{{WRecall}^{-1}+{WPrecision}^{-1}}\right),$$

(16) $$False\;Alarm\;Rate\;\left(FAR\right)=\frac{\sum_{j\in S,\;j\neq 0}n_{0j}}{n_{0}}.$$

Here, the FAR is the proportion of normal traffic incorrectly flagged as an attack (i.e., false alarms on the normal class).

We use *novel attack detection rate* (*NADR*) and *novel attack false alarm rate* (NAFAR) metrics to evaluate the performance of GR-IDS for detecting novel attacks. Let k denote the class ID of an *unseen* attack. These metrics are defined as

(17) $$NADR\left(k\right)=Recall\left(k\right)=\frac{n_{kk}}{n_{k}},$$

(18) $$NAFAR\left(k\right)=\frac{\sum_{i\in S,\;i\neq k}n_{ik}}{n-n_{k}},$$

### 4.3. Experimental Setup

GR-IDS is implemented using the PyTorch 2.1 deep learning library and Python 3.10. The preprocessing steps of the datasets include the following: (1) removing duplicated flows, (2) encoding the categorical features using label encoding, (3) filtering out sessions that contain fewer than *five packets*, and (4) *under-sampling* the majority classes to alleviate class imbalance. We apply a *one-sided selection strategy* for any class containing more than 10,000 sessions.

In the experiments, we split the available data into a training/test set with ratios of 70% and 30%, respectively. To optimize the hyperparameters of GR-IDS, we use *5-fold cross-validation* and the *random search* strategy. The search space of the hyperparameters is specified in Table 4. Additionally, the architectures of the transformer and LSTM networks are specified in Table 2.

### 4.4. Early Detection Performance

This section aims to estimate the required number of packets of an ongoing session for reliable classification. Let the parameter $N_{p}$ determine the number of *initial* consecutive packets from a session used for intrusion detection. $N_{p}$ must be large enough to capture the temporal patterns of various attacks while also remaining small enough to allow for near-real-time detection.

To identify appropriate values for $N_{p}$, we set up multiple experiments on the CIC_DDoS2019 dataset. In the i-th experiment $\left(i\in \left[10\right]\right)$, we only use i *initial* packets of each session for training and testing. For each experiment, we optimize the values of other hyperparameters in the ranges specified in Table 3. Specifically, we optimize the hyperparameter M (sequence length) from the range {3, 4, 5}. Table 5 summarizes the distribution of the number of packets per session in this dataset.

The performance of GR-IDS in these experiments is presented in Table 6. The results indicate that as the number of processed packets in sessions increases, the detection rate improves. Considering only a few initial packets of a session yields a poor detection rate. This indicates that these packets often do not contain malicious patterns as they are used to establish a connection in TCP sessions and rarely carry attack signatures. For example, our method obtains 78.97% accuracy and an 18.56 FAR by setting $N_{p}=1$. However, GR-IDS achieves 90.09% accuracy and a 7.23% FAR by processing four packets.

Moreover, GR-IDS achieves an accuracy of 97.47% and a FAR of 0.4% by setting $N_{p}=7$. The improvement in the results was not significant for larger values of $N_{p}$. The results confirm that GR-IDS can detect attack activities early by processing fewer than seven packets of a session while maintaining a low FAR, which indicates a remarkable improvement compared to session-based methods that need the full session (≈30 packets on average) to detect intrusions.

### 4.5. Detection Performance

This section evaluates the performance of GR-IDS for detecting known (observed) attack types. To this end, we conduct two experiments on the CIC_DDoS2019 and CRiSIS-2022 datasets.

In these experiments, we set $N_{p}=7$ for both GR-IDS and the method of Han et al. [1], and $N_{p}=9$ for SPIN-IDS [33] as recommended by its authors.

**Figure 3:**
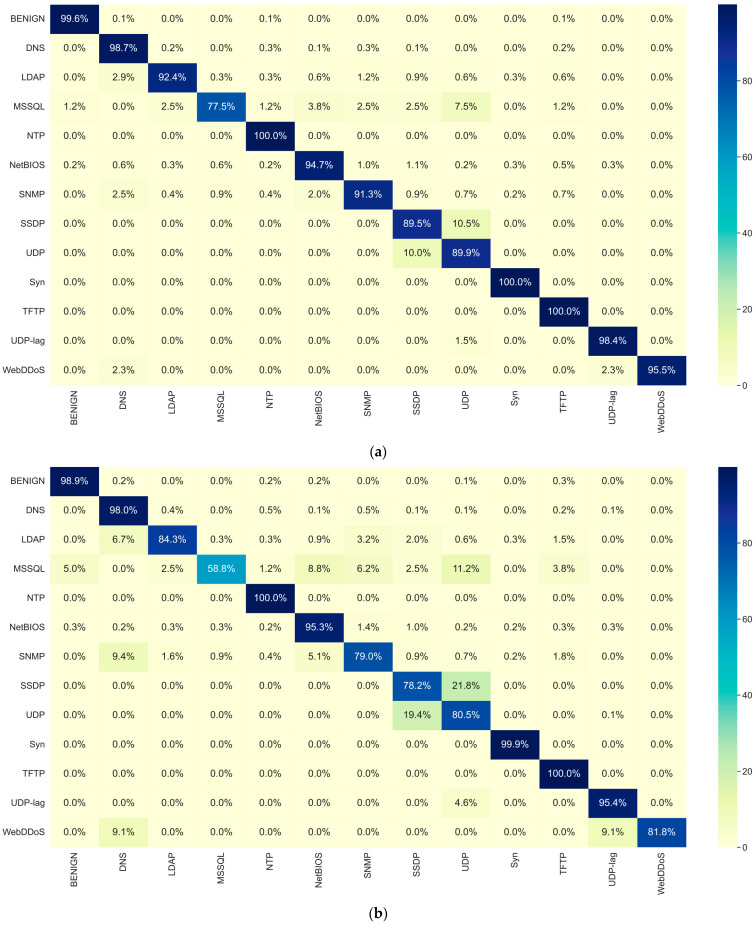
Comparison between (**a**) confusion matrix of GR-IDS and that of (**b**) SPIN-IDS on CIC_DDoS2019.

Table 7 presents the multi-class classification results of GR-IDS and several state-of-the-art methods on CIC_DDoS2019, including both packet-based and session-based approaches. Moreover, Figure 3 compares the confusion matrix of GR-IDS with SPIN-IDS, providing a better insight into the classification results.

The results confirm that GR-IDS extracts discriminative features from sessions and surpasses all the competing methods by a large margin across all classification metrics. Specifically, in the *normal* class, misclassification rates of GR-IDS are 0.1%→DNS, 0.1%→NTP, and 0.1%→TFTP; all other attack types receive 0.0% FPs (rounded at 0.05%). Moreover, in classifying attack types, only 1.2% and 0.2% of examples of MSSQL and NetBIOS are misclassified as normal; all remaining attack types show 0.0% FNs.

Additionally, GR-IDS attains an accuracy of 97.47% and FAR of 0.4%, while SPIN-IDS achieves an accuracy of 94.92% and FAR of 1.09%.

The balanced performance of GR-IDS is considerably higher than other methods. GR-IDS achieves macro accuracy of 94.43%, surpassing SPIN-IDS by a large improvement of 5.96%. This indicates that GR-IDS classifies all classes consistently well, even minority classes such as WebDDoS and LDAP.

We also evaluated the performance of GR-IDS across different session sizes on CIC_DDoS2019. To this end, we categorized the sessions into four categories based on the number of packets (small: ≤10, M: 11–30, L: 31–100, XL: >1000). Table 8 compares the results of GR_IDS and SPIN-IDS in these categories.

The results indicate that the performance of our method is stable across different session sizes. For example, the minimum and maximum accuracies of GR-IDS fall between 96.75% and 99.93% across these categories. Moreover, the FAR values remain less than 0.63% over all of them. Additionally, GR-IDS consistently outperforms SPIN-IDS at different session sizes.

The multi-class classification results of GR-IDS and the competing methods on CRiSIS-2022 are presented in Table 9. These results align with previous results on CIC_DDoS2019.

GR-IDS achieves the best performance in this experiment with an accuracy of 99.90% and a low FAR of 0.11%. Moreover, the balanced performance of GR-IDS is remarkable, with macro accuracy of 92.60%. The high balanced performance of GR-IDS is mainly attributed to the proposed loss function, the multi-proxy per-class mechanism, and the proposed adaptive class-wise thresholds. These result in a robust embedding in which classes are well separated despite imbalanced distributions.

### 4.6. Identifying Novel Attacks

We also conduct several experiments to evaluate the performance of GR-IDS in detecting novel attacks. For this purpose, we first group classes in CRiSIS-2022 into six categories as shown in Table 10. In each experiment, one attack category is excluded from the training set and GR-IDS is trained in the remaining categories. During test phase, we assess GR-IDS on the test set of CRiSIS-2022, which includes all attack categories (both the unobserved category and the observed categories).

The performance of GR-IDS is compared with several *out-of-distribution detection* (OOD) methods introduced in [49] using the NADR and NAFAR metrics. The OOD methods are denoted by *confidence* (CONF), *Monte Carlo Dropout* (MCD), *K-Nearest Neighbor* (KNN), and *Silhouette measure* (SLH).

The competing methods require a predefined threshold to identify novel intrusions. We adjust these thresholds using the common technique, which guarantees that 95% of the validation data are classified as one of the observed classes [50]. However, GR-IDS uses the proposed class-wise thresholds as described in Section 3.4.

We adjust the hyperparameters of OOD methods according to the recommended settings in [49]. Specifically, we set the learning rate = 0.0005, batch size = 512, number of epochs = 25, and optimizer = Adam for these methods. These methods are trained using cross-entropy and a center loss (with a weighting factor of 1). Also, the model with the highest F1-score on the validation set is used for testing. Table 11 presents the results.

As the results indicate, GR-IDS surpasses the other methods by a large margin. For example, it detects the unobserved Brute Force attack with an accuracy of 99.76% and a low NAFAR of 2.23%, whereas the best OOD method, kNN, attains an accuracy of 94.38% with a higher NAFAR of 6.02%. Furthermore, while all OOD methods fail to identify the novel Botnet ARES intrusion, GR-IDS detects this attack with a NADR of 99.21% and a relatively low NAFAR of 5.75%.

It should be noted that all competing methods in this experiment are session-based and thus must wait for 57.29 packets (on average) to detect intrusions. In contrast, GR-IDS can accurately detect intrusions by processing only the first seven packets of a session.

### 4.7. Ablation Study

This section examines the contribution of several modules and mechanisms within GR-IDS to the overall performance of our method. Specifically, we examine the effects of statistical features, the LSTM network, the compactness loss term, and the proposed adaptive class-wise thresholds. To this end, we derive four variants of GR-IDS as follows:

1. *GR-IDS w/o SF:* Obtained by removing the statistical features from the feature encoder.
2. *GR-IDS w/o LSTM:* Derived by omitting the LSTM network from the feature encoder.
3. *GR-IDS w/o C:* Indicates GR-IDS without the compactness loss term.
4. *GR-IDS w/o C-Thresh:* Shows GR-IDS without the adaptive class-wise thresholds.
5. *GR-IDS Pure Trans:* Shows a GR-IDS where the feature encoding module is replaced by a transformer.
6. *GR-IDS Pure LSTM:* Indicates a GR-IDS where the feature encoding module is replaced by an LSTM.

These variants are evaluated for detecting observed intrusions on CIC_DDoS2019 and novel intrusions on CRiSIS-2022. We preprocess the CIC_DDoS2019 and CRiSIS-2022 datasets as specified in Section 4.3 and Section 4.6, respectively.

**Figure 4:**
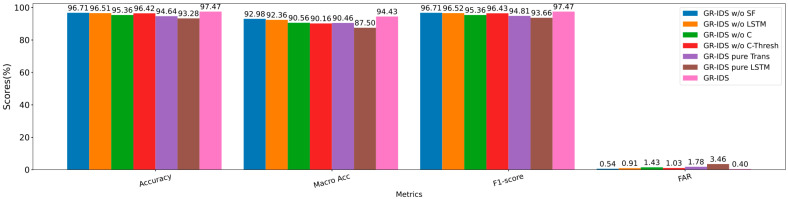
Multi-class classification results of the ablation study on CIC_DDoS2019.

The detection performance of these variants on CIC_DDoS2019 is illustrated in Figure 4. Additionally, Table 12 presents the novel attack performance of these methods.

The results indicate that all these modules and mechanisms enhance the performance of GR-IDS. Moreover, the considerable performance gains of the full GR-IDS with respect to the simpler feature encoder variants (GR-IDS *Pure Trans* and *GR-IDS Pure LSTM*) justify the effectiveness of the proposed feature encoder.

Without the statistical features, we observe that GR-IDS’s performance is slightly reduced. However, the overall and balanced performance of GR-IDS is still high.

The LSTM network has a more positive effect than statistical features. Our offline analysis shows that this network is especially beneficial for processing long sessions. However, 50% of sessions in CIC_DDoS2019 contain less than six packets, as shown in Table 5. Moreover, we only process seven initial packets of any session for early detection. Thus, the effect of this network is not considerable.

The compactness loss positively affects both overall and balanced performance of GR-IDS. Without this loss term, the accuracy and macro accuracy of GR-IDS reduce by 2.26% and 2.83%. Moreover, the FAR of GR-IDS increases from 0.4% to 1.43% in this case. Therefore, we conclude that this term is necessary for maintaining high performance in GR-IDS.

Finally, the proposed adaptive class-wise threshold mechanism slightly increases the overall performance of GR-IDS. However, this mechanism is very effective for obtaining a high balanced performance. For example, without this mechanism, the accuracy and F1-score of GR-IDS are reduced by around 1%. However, the macro accuracy and macro F1-score of GR-IDS are reduced by 4.27% and 3.65%, respectively.

The results in Table 12 show that statistical features and the LSTM network slightly increase the novel attack detection performance. Conversely, the compactness loss and the proposed adaptive class-wise thresholds highly contribute to the novel attack detection rate. For example, without one of these features, the NADR of *GR-IDS* decreases by around 4%.

We can conclude that the compactness loss results in compact representations for each class in the embedding space which, in turn, yields a highly balanced and generalized performance for GR-IDS. Additionally, the proposed adaptive class-wise threshold mechanism successfully mitigates the imbalance issue and bias toward observed classes in NIDSs. Moreover, statistical features and the LSTM network consistently enhance the performance of GR-IDS, although their improvements are slight.

### 4.8. Hyperparameter Analysis

This section investigates two hyperparameters of GR-IDS: J (number of proxies per class) and M (packet sequence length). To this end, we conduct two experiments on CIC_DDoS2019.

**Figure 5:**
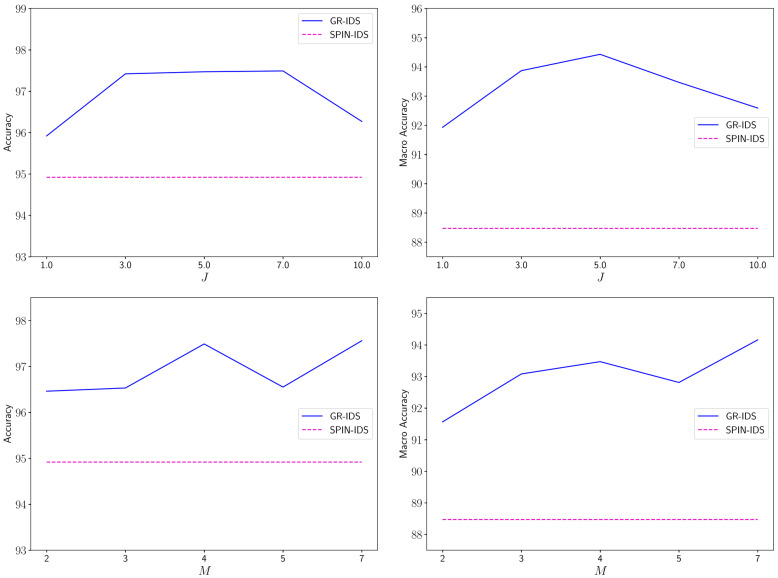
Change in accuracy and macro accuracy of GR-IDS by varying J (first row) and M (second row) on the CIC_DDoS2019 dataset.

In the first experiment, we change the value of J in the range $\left\{1,3,5,7,10\right\}$ and measure the accuracy and macro accuracy of GR-IDS within this range. Figure 5 plots the results. To provide better insight into the sensitivity of this hyperparameter, we also illustrate the results of SPIN-IDS.

As the results show, the performance of GR-IDS consistently improves as J increases. Considering both accuracy and macro accuracy metrics, the best performance is obtained by setting J=5. For larger values of J, the performance of GR-IDS slightly decreases. At the optimal point, the accuracy and macro accuracy are around 1.5% and 2% higher than those obtained by J=1. This indicates the effectiveness of considering multiple proxies per class in NIDSs, as this technique better captures the high intra-class variability in this domain, especially for larger classes. In addition, GR-IDS outperforms SPIN-IDS for a wide range of J values, which indicates the low sensitivity of the performance to a specific value of J.

In the second experiment, the accuracy and macro accuracy of GR-IDS are measured as M varies in the range $\left\{2,3,4,5,7\right\}$. The results are illustrated in Figure 5.

As the results indicate, the performance of GR-IDS improves by increasing M. The best performance of GR-IDS is achieved by setting M=7. Interestingly, we observe that the results for M=5 are slightly lower than M=4.

The reason is that we limit the number of processed packets of each session to seven. Thus, for M=5, the transformer processes five packets first and two packets subsequently. The imbalance between the two windows slightly reduces the performance of the feature encoder. Additionally, the results confirm that GR-IDS has less sensitivity to a specific value of M.

## 5. Conclusions and Future Work

This research presents an incremental and similarity learning method called GR-IDS for *early* and *multi-class* intrusion detection. GR-IDS includes an *incremental* feature encoding module that effectively captures temporal and spatial information between the packets of a session. Simultaneously, GR-IDS incrementally computes statistical features of the session. By combining these features and learning a discriminative embedding space through the proposed multi-proxy loss, GR-IDS can detect network intrusions early using only initial packets of a session with high performance.

Additionally, by utilizing a compactness loss term and a novel class-wise thresholding technique, it can accurately identify novel or mutated attack types.

Several experiments were conducted to evaluate the performance of GR-IDS for detecting observed and unobserved attack types. The results were also compared with several state-of-the-art methods. The results indicate that GR-IDS can effectively detect attack activities by processing fewer than seven packets of a session. Moreover, GR-IDS surpasses all the competing methods by a large margin in terms of many classification metrics.

The results of novel attack detection show that GR-IDS identifies various unobserved attack types with a higher detection rate compared to peer methods, while having a lower false novel attack alarm rate. For example, it detects the unobserved Brute Force attack with an accuracy of 99.76% and a low NAFAR of 2.23%, whereas the best *out-of-distribution* (OOD) method achieves an accuracy of 94.38% and a NAFAR of 6.02%.

The ablation study validates the effectiveness of all evaluated components in the proposed method. Specifically, the results confirm that the compactness loss term and the proposed class-wise thresholding technique considerably improve both overall and balanced performance of GR-IDS.

Currently, we focus on training and evaluation on existing datasets. However, validating our work on a real or virtual network with live attacks would provide a more comprehensive assessment of real-world performance.

In future research, we plan to set up scenarios that mirror the characteristics of benchmark attacks and report the detection results to quantify any performance difference between dataset-based and emulated evaluations. We will also extend GR-IDS to identify adversarial attacks and extend the generalizability of our method across different datasets.

## Figures and Tables

**Table 1:** Statistical features and variables for each session. Here, ✓ indicates that the variable is used in that role (either as a feature or stored in session state).

Variable (s)	Description	Update Eq.	Feature	State
last_IAT	Last IAT in the session	last_IAT←t−last_TS		✓
last_TS	Timestamp of the last packet in the session	last_TS←t		✓
n	Number of packets in the session	n←n+1		✓
len	Length of the session in bytes	len←len+l		✓
duration	Duration of the session in seconds	duration←duration+last_IAT		✓
pkt_rate	Packet rate per second in the session	$pkt\_rate=\frac{n}{duration}$	✓	
$\bar{IAT}$, IAT_std	Mean and standard deviation of IATs in the session	$\bar{IAT}\leftarrow \frac{1}{n}\left(\bar{IAT}\times \left(n-1\right)+last\_IAT\right)$ $IAT\_std=\sqrt{\bar{IAT^{2}}-{\left(\bar{IAT}\right)}^{2}}$	✓	✓
IAT_max	Maximum of IATs in the session	$IAT\_max\leftarrow \max\left\{IAT\_max,last_{I}AT\right\}$	✓	✓
$\bar{IAT^{2}}$	Mean of the squares of IATs in the session	$\bar{IAT^{2}}\leftarrow \left(\bar{IAT^{2}}\times \left(n-1\right)+{\left(last\_IAT\right)}^{2}\right)$		✓
$\bar{pkt\_len}$, pkt_len_std	Mean and standard deviation of packet lengths in the session	$\bar{pkt\_len}\leftarrow \frac{1}{n}\left(\bar{pkt\_len}\times \left(n-1\right)+l\right)$ $pkt\_len\_std=\sqrt{\bar{{pkt\_len}^{2}}-{\left(\bar{pkt\_len}\right)}^{2}}$	✓	✓
$\bar{{pkt\_len}^{2}}$	Mean of the squares of packet lengths in the session	$\bar{{pkt\_len}^{2}}\leftarrow \frac{1}{n}\left(\bar{{pkt\_len}^{2}}\times \left(n-1\right)+l^{2}\right)$		✓

**Table 2:** Specification and architecture of the transformer and LSTM networks.

Layer/Block	Input	Output	Connected To	#Parameters
Transformer ^1^				
FC1 ^2^ + Pos Emb	Md	$Md_{T}$	Input	$dd_{T}+d_{T}$
$TB_{1}\;{\left(h=4\right)}^{\;3}$	$Md_{T}$	$Md_{T}$	FC1+ Pos Emb	$d_{T}^{2}+d_{T}$
$TB_{2}\left(h=4\right)$	$Md_{T}$	$Md_{T}$	$TB_{1}\left(h=4\right)$	$d_{T}^{2}+d_{T}$
FC2	$Md_{T}$	$Md_{T}$	$TB_{2}\left(h=4\right)$	$d_{T}^{2}+d_{T}$
FC3	$Md_{T}$	$Md_{T}$	FC2	$d_{T}^{2}+d_{T}$
LSTM	$Md_{T}$	$d_{T}$	FC3	$(4M+1)d_{T}^{2}+4d_{T}$

^1^ ${\;d}_{T}$ : Transformer dimension. ^2^ FC: Fully connected. ^3^ TB: Transformer block.

**Table 3:** Statistics of the NIDS datasets in our experiments.

Dataset	#Features	#Flows	#Classes	Categories	Description
CIC_DDoS2019	80	4,534,059	13	Benign and twelve different DDoS attacks	Recorded during two days of network activity, containing both extracted flow features and raw packet sequences.
CRiSIS-2022	79	2,830,743	15	Benign and fourteen different attacks	Corrected version of CIC-IDS2017 that includes flow features and pcap files.

**Table 4:** Hyperparameters of GR-IDS and their search spaces.

Hyperparameters	Description	Search Space
lr	Learning rate	$\left\{0.1,\;0.3,\;0.5,\;0.7,\;1,\;3,\;5\right\}\times 10^{-3}$
B	Batch size	$\left\{64,\;128,\;256,\;512\right\}$
opt	Optimizer algorithm	Adam
m	Margin in the compactness loss term	0.1, 0.3, 0.5, 1
M	Sequence length	3, 4, 5
J	Number of proxies per class	1, 3, 5, 7, 10
$d_{e}$	Embedding dimension	32

**Table 5:** Statistics of number of packets per session within each class on CIC_DDoS2019.

Class	#Sessions	Mean	Std	Min	25%	50%	75%	Max
BENIGN	14,944	39.39	131.6	5	8	17	49	7526
DrDoS_DNS	13,154	1548.63	11,591.99	5	174	200	200	100,150
DrDoS_LDAP	1147	4798.67	19,708.64	5	8	8	8	86,232
DrDoS_MSSQL	266	1529.99	5127.89	5	6	15	37.5	61,421
DrDoS_NTP	1,088,415	70.24	63.67	5	20	46	104	400
DrDoS_NetBIOS	2077	12.94	95.72	5	8	8	8	4308
DrDoS_SNMP	1494	5168.54	21,053.86	5	8	10	16	92,128
DrDoS_SSDP	496,972	7.93	70.73	5	6	6	6	49,690
DrDoS_UDP	604,806	7.96	7.5	5	6	6	6	2430
Syn	131,317	14.94	18.41	5	12	14	18	4692
TFTP	2,126,468	7.67	4.32	5	6	6	8	664
UDP-lag	52,852	7.97	63.01	5	6	6	8	8666
WebDDoS	147	16.14	6.51	5	15	15	21	63
**Total**	4,534,059	30.55	811.89	5	6	6	16	100,150

**Table 6:** Detection performance of GR-IDS versus $N_{p}$ on CIC_DDoS2019.

$N_{p}$	Accuracy	Macro Acc	Precision (DR)	F1-Score	Macro F1-Score	FAR
1	78.97	75.36	88.60	82.99	40.15	18.56
2	84.91	80.56	91.08	87.54	44.72	12.13
3	87.97	83.72	92.41	89.87	47.87	9.35
4	90.09	87.31	93.44	91.52	50.64	7.23
5	93.08	89.48	94.83	93.81	56.34	4.1
6	96.24	92.57	96.54	96.36	71.54	1.49
7	97.47	94.43	97.48	97.47	94.14	0.4
8	97.49	94.63	97.5	97.49	95.51	0.00
9	97.49	93.28	97.49	97.49	93.68	0.54
10	97.56	95.09	97.57	97.57	95.39	0.00
11	97.58	95.41	97.59	97.58	94.63	0.47
12	97.56	94.18	97.56	97.56	93.78	0.36

**Table 7:** *Multi-class classification* performance (%) of the competing NIDSs on CIC_DDoS2019.

Type	Method	Accuracy	Macro Acc	Precision (DR)	F1-Score	Macro F1-Score	FAR
Session-based	Yang et al. [47]	93.36	84.67	93.38	93.37	85.51	2.25
	DBN [48]	91.13	80.25	91.28	90.68	91.13	0.02
Packet-based	Han et al. [1]	92.25	84.22	92.27	92.26	84.26	2.12
	SPIN-IDS [33]	94.92	88.47	94.93	94.92	89.00	1.09
	GR-IDS	97.47	94.43	97.48	97.47	94.14	0.40

**Table 8:** *Multi-class classification* performance (%) across different session sizes on CIC_DDoS2019.

Session Size	Method	Accuracy	Macro Acc	Precision (DR)	F1-Score	Macro F1-Score	FAR
Small	GR-IDS	**96.75**	**94.30**	**96.76**	**96.75**	**92.50**	**0.63**
	SPIN-IDS [33]	93.21	88.05	93.23	93.22	87.79	1.40
Medium	GR-IDS	**98.03**	**94.88**	**98.04**	**98.03**	**94.55**	**0.30**
	SPIN-IDS	96.05	88.67	96.08	96.05	87.56	1.01
Large	GR-IDS	**99.94**	**96.67**	**99.94**	**99.94**	**96.13**	**0.22**
	SPIN-IDS	99.87	84.68	99.88	99.87	83.56	1.03
XLarge	GR-IDS	**99.93**	**96.27**	**99.94**	**99.93**	**88.13**	**0.00**
	SPIN-IDS	99.87	84.68	99.88	99.87	83.56	1.03

**Table 9:** *Multi-class classification* performance (%) of the competing NIDSs on CRiSIS-2022.

Type	Method	Accuracy	Macro Acc	Precision (DR)	F1-Score	Macro F1-Score	FAR
Session-based	Yang et al. [47]	99.81	87.50	99.35	99.34	90.37	0.33
	DBN [48]	94.19	63.39	99.58	96.73	48.78	3.97
Packet-based	Han et al. [1]	99.67	84.20	99.69	99.68	83.13	0.40
	SPIN-IDS [33]	99.10	88.73	99.12	99.11	84.26	0.40
	GR-IDS	**99.90**	**92.60**	**99.90**	**99.90**	**91.23**	**0.11**

**Table 10:** Categories and classes of CRiSIS-2022.

Category	Classes	#Sessions
BENIGN	BENIGN	432,946
Botnet	Botnet	738
DDoS	DDoS, DoS GoldenEye, DoS Hulk, DoS Slowhttptest, DoS slowloris, Heartbleed	265,308
Brute Force	FTP-Patator, SSH-Patator	6953
PortScan	PortScan	221
Web Attack	Web Attack-Brute Force, Web Attack-Sql Injection, Web Attack-XSS	190
**Total**		706,356

**Table 11:** Novel attack detection performance (%) of the competing methods on CRiSIS-2022.

Novel Attack	GR-IDS NADR/NAFAR	CONF NADR/NAFAR	MCD NADR/NAFAR	KNN NADR/NAFAR	SLH NADR/NAFAR
Web Attack	**99.71/4.01**	93.10/4.97	93.10/6.16	90.30/5.69	88.58/1.46
PortScan	**62.64/3.03**	5.28/4.99	38.64/5.96	40.26/6.02	1.23/1.53
DoS/DDoS	**59.2/3.15**	26.95/4.92	28.98/5.79	30.25/6.15	13.65/1.63
Brute Force	**99.76/2.23**	2.15/4.95	76.02/5.75	94.38/6.02	1.85/1.43
Botnet ARES	**99.21/5.75**	0.00/5.00	3.77/6.21	0.00/5.17	0.00/1.12
**AVG**	**84.10/3.63**	25.50/4.97	48.10/5.97	51.04/5.81	21.50/1.43

**Table 12:** Ablation study results for novel attack detection performance (%) on CRiSIS-2022.

Novel Attack	GR-IDS NADR/NAFAR	w/o LSTM NADR/NAFAR	w/o C NADR/NAFAR	w/o C-Thresh NADR/NAFAR	Pure Trans NADR/NAFAR	Pure LSTM NADR/NAFAR
Web Attack	**99.71/4.01**	98.23/4.46	96.56/5.81	96.79/5.66	98.27/3.17	95.68/5.08
PortScan	**62.64/3.03**	61.56/2.78	59.82/3.03	59.98/3.99	59.9/6.17	59.21/3.77
DoS/DDoS	**59.20/3.15**	58.49/3.82	55.26/4.58	55.51/4.51	56.91/3.89	55.84/3.49
Brute Force	**99.76/2.23**	98.19/3.03	95.84/3.40	94.65/3.03	96.73/0.6	93.93/3.55
Botnet ARES	**99.21/5.75**	98.79/6.31	94.72/6.88	94.5/5.99	95.39/7.4	93.53/6.98
**AVG**	**84.10/3.63**	83.05/4.05	80.44/4.74	80.29/4.64	81.64/4.25	79.64/4.57

## Data Availability

The datasets used in the experiments are publicly available and can be downloaded from the following links: CIC_DDoS2019 dataset: https://www.unb.ca/cic/datasets/ddos-2019.html (accessed on 5 August 2025). CRiSIS-2022 dataset: https://gitlab.inria.fr/mlanvin/crisis2022 (accessed on 5 August 2025).
